# Cost and efficiency benefits of a single modality imaging strategy for pre-procedural workup of AF catheter ablation

**DOI:** 10.1186/1532-429X-17-S1-P230

**Published:** 2015-02-03

**Authors:** Pranav Bhagirath, Elise van Dongen, Maurits van der Graaf, Suresh Ghoerbien, Vincent J van Driel, Hemanth Ramanna, Marco Götte

**Affiliations:** Cardiology, HAGA Teaching Hospital, The Hague, Netherlands

## Background

Multimodality imaging is considered a cornerstone in the workup of catheter ablation for atrial fibrillation (AF). Advances in imaging modalities suggest a single modality approach could prove as effective as the current multimodality strategy. This could simplify the imaging workflow and potentially reduce financial and time burden upon the healthcare system.

## Methods

A literature review was performed to identify frequently used parameters for patient assessment prior to catheter ablation of AF. Subsequently, the role of four key non-invasive imaging modalities in performing this assessment was examined.

A cost and time analysis was performed for two conventional multimodality imaging approaches and a cardiac magnetic resonance (CMR) only strategy. The cost of examination was according to the Netherlands standard care between July-2013 and December-2013.

## Results

**Figure 1:**
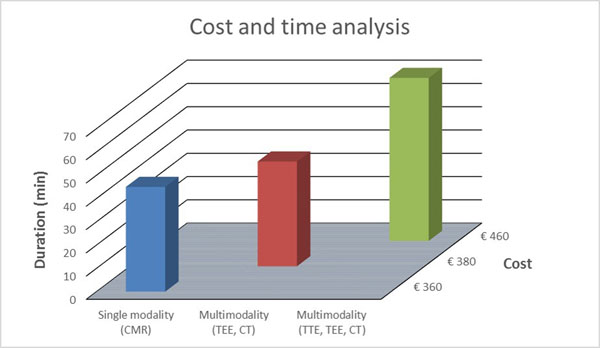
Cost comparison between single-modality and multimodality patient workup.

Five parameters (three key and two optional) were identified in the workup of catheter ablation (Table 1). Out of the 4 key imaging modalities, CMR provided the highest diagnostic yield and enabled a complete coverage of both key and optional parameters (Table 1). The total cost of imaging ranged from €360 to €460 per patient (Figure 1). The multimodality approaches were up to 22% more expensive and 35% more time consuming compared to the CMR only strategy (Figure 1).

**Table 1:** Comparison between imaging modalities for providing key and optional parameters required during catheter ablation workup.

	TTE	TEE	CT	CMR
LA dimensions (key)	+	++	++	++
LA fibrosis (optional)				+
LA geometry (optional)			+	+
LAA thrombus (key)		+	+	+
PV anatomy (key)		+	+	+

## Conclusions

A CMR based imaging approach for pre-procedural workup of AF ablation is:

1. Comprehensive, providing all parameters required to perform a thorough assessment.
2. Cost-efficient, saving up to €100 per patient in comparison to conventional strategies.
3. Time efficient, acquiring all information in a single examination compared to the current (often fragmented) approach.

